# Recollection-Based Retrieval Is Influenced by Contextual Variation at Encoding but Not at Retrieval

**DOI:** 10.1371/journal.pone.0130403

**Published:** 2015-07-02

**Authors:** Eyal Rosenstreich, Yonatan Goshen-Gottstein

**Affiliations:** Tel-Aviv University, Ramat Aviv, Israel; University of Akron, UNITED STATES

## Abstract

In this article, we investigated the effects of variations at encoding and retrieval on recollection. We argue that recollection is more likely to be affected by the processing that information undergoes at encoding than at retrieval. To date, manipulations shown to affect recollection were typically carried out at encoding. Therefore, an open question is whether these same manipulations would also affect recollection when carried out at retrieval, or whether there is an inherent connection between their effects on recollection and the encoding stage. We therefore manipulated, at either encoding or retrieval, fluency of processing (Experiment 1)—typically found not to affect recollection—and the amount of attentional resources available for processing (Experiments 2 and 3)—typically reported to affect recollection. We found that regardless of the type of manipulation, recollection was affected more by manipulations carried out at encoding and was essentially unaffected when these manipulations were carried out at retrieval. These findings suggest an inherent dependency between recollection-based retrieval and the encoding stage. It seems that because recollection is a contextual-based retrieval process, it is determined by the processing information undergoes at encoding—at the time when context is bound with the items—but not at retrieval—when context is only recovered.

## Introduction

Memory processes are often characterized by the nature of the manipulations affecting them. For example, the demonstration that certain tests of memory are affected by a levels-of-processing manipulation has been used as evidence for the semantic/ conceptual nature of memory [1] [2] [3] [4]. Guided by this notion, in three experiments described in this article, we suggest that a more accurate understanding of the processes underlying memory performance may be achieved by considering the stage in which these manipulations are carried out (i.e., encoding versus retrieval). Consequently, we present a modified interpretation for the nature of the processes underlying recognition—namely recollection and familiarity—which focuses on memory stage as an important factor in predicting them.

Over the past 40 years, numerous recognition-memory studies examined the effects of a variety of manipulations on memory performance. For example, deeply processed items, such as to-be-solved anagrams of words presented at study, were more likely to be remembered than shallowly-processed items, such as the words in their standard form (i.e., a levels-of-processing effect; [5] [6]). Similar patterns were observed using full- versus divided-attention manipulations (e.g., [7]).

In addition, several demonstrations showed that manipulating the speed, or fluency, at which an item was processed, increased the probability that the item would subsequently be judged as studied. For example, Whittlesea [8] [9] (see also [10]) presented participants with a list of to-be-remembered words at study. At test, both studied and unstudied words were presented as terminal words in two types of sentences. In one type, the sentence predicted the target word (i.e., The stormy sea tossed the ***BOAT***; the *fluent condition*). In the second type of sentence, the sentence did not predict the target word (i.e., She saved her money and bought a **BOAT**; the *non-fluent condition*). Results revealed that words presented in the fluent condition were more likely to be judged as studied, as compared to words presented in the non-fluent condition, thereby producing both more hits and more false alarms (FAs). Whittlesea explained this effect by an automatic, heuristic, misattribution of the fluent processing of the terminal word to a previous encounter with the target word. Indeed, when participants were aware of purpose of the fluency manipulation, no effect was found [11] [12].

The manipulations that have been found to affect recognition memory can be roughly classified into two categories (cf., the fluency-distinctiveness model [13]). The first category consists of procedures designed to manipulate the extent to which an item or its meaning are processed. This category includes manipulations as the Read-Anagram (in which words are presented in their normal form as compared to scrambled. For example, TABLE vs. ELBAT), Read-Generate (in which words are read as compared to generated. For example, TABLE vs. TA__E) and Divided Attention (in which words are studied without performing a secondary task, as compared to studied while performing a different task). The second category consists of manipulations designed to control the speed at which information is processed, with research programs reflecting Whittlesea’s fluency manipulation. This category also includes priming manipulations [14] [15], known to influence processing times, and hence, predicted to affect the fluency of processing.

Interestingly, the classification of the different manipulations into two categories elegantly maps onto the two types of subjective experience that participants are able to associate with the recognition of an item. The first type of subjective experience entails remembering contextual information related to the target item. The second type entails knowing that the target item was studied, in the absence of conscious recollection of context detail [16] [17] (for a similar idea in free recall, see [18]). One way to tap these two different processes is the Remember-Know (RK) task ([17]; other techniques include dual-process ROC curves and the process-dissociation paradigm), wherein participants are instructed to respond “Remember” (R) if they judge an item as studied because they have clear recollection of the item and its study context. In contrast, participants are asked to respond “Know” (K) if they judge an item as studied without the recollection of episodic features from the study stage.

Critically, it has been demonstrated that manipulations belonging to the first category (manipulating the extent of item's processing) primarily affect R responses whereas those belonging to the second category (manipulating the speed of item's processing) primarily affect K responses (cf., [13]). To illustrate, Yonelinas reported that a divided-attention manipulation at study affected the proportion of R responses, yet hardly affected the proportion of K responses [19]. Similarly, promoting deeper processing of words at study yielded higher rates of R as compared to shallow processing [20]. In contrast, preceding the presentation of a test item (i.e., the recognition probe) with a semantically-related item (e.g., study: "NURSE"; Test: "doctor- NURSE"; Semantic-priming paradigm), yielded higher proportion of K responses as compared to preceding the item with an unrelated item (e.g., study: "NURSE"; Test: "phone- NURSE"). This manipulation, however, did not affect the proportion of R responses [21] [22].

Different models of recognition have been proposed to explain the way in which different manipulations affect recognition memory, with a particular focus on the RK procedure. Single-process models explain the effect of different manipulations by a shift in a subjective decision criterion located on a hypothetical axis of memory strength (or mnemonic evidence), with different levels of the manipulation producing different levels of strength of the memory trace [23] [24] [25]. Accordingly, the R and K responses reflect the different levels of strength, with R responses reflecting higher levels of strength than K responses.

In contrast, dual-process models of recognition memory postulate that two distinct processes underlie retrieval from recognition memory, Recollection and Familiarity. Based on behavioral data [21] [26] [27] [28] [29] [30] and on a neuropsychological double dissociations between recollection and familiarity[31] [32] [33] [34], it has been argued that recollection and familiarity do not index the retrieval of different levels of strength, but rather the retrieval of different forms of memory. For sake of simplicity, the studies in this article are described in terms of an influential version of dual-process model [27] [29] [35].

According to Yonelinas, recollection entails the conscious retrieval of an event including contextual detail related to the event. Familiarity, in contrast, is considered to be a non-contextual, automatic, retrieval process that induces the feeling that an event was encountered before [27] [35] (see [36], for a comprehensive review of these and other differences between the two processes). If so, the nature of the manipulation carried out should be a major determinant of the memory process affected by the manipulation. Thus, manipulations designed to affect the depth to which an item was processed—or in other words, the extent to which contextual information is available—should affect only recollection but are not likely to affect familiarity. In contrast, manipulations designed to induce some sort of unconscious sensation that could be attributed to past occurrence, should affect familiarity but not recollection.

Here, our goal was to further understand how recollection and familiarity are affected by different manipulations. Our thesis is based on the straightforward notion that the process of recollection entails retrieving information that was part of the encoding context. Because encoding is the memory stage in which context is bound with the item[37] [38] [39]; and because the process of recollection is intimately related to the encoding context, it follows that manipulations undertaken at encoding should affect recollection and that the identical manipulations should not affect recollection when carried out at retrieval. Thus, we argue that, irrespective of the characteristics of the manipulation, a major predictor for when recollection should be affected is the stage at which the manipulation is carried out, encoding or retrieval.

In reviewing the literature, we have not found any suggestion that knowledge of the memory stage (encoding, retrieval) in which a manipulation was carried out can largely predict performance—irrespective of other details regarding the design, procedure and the, most importantly, the nature of the manipulation. Instead, we found two alternative proposals for predictors of performance. The first proposal, as described above, focuses on the nature of the manipulation (e.g., directing participants' attention to the processing of the item or to its meaning as compared to manipulations designed to affect the speed of processing; cf., [13]). The second proposalfocuses on study-test compatibility, highlighting the importance of such compatibility to recollection [40]. While not pitting these suggestions with ours, here we endorse the contribution of a third, hitherto, ignored variable—memory stage.

Thus, we argue that memory-stage may be a helpful construct for understanding the variability found in the literature on differential effects on recollection and familiarity by numerous variables. Specifically, we suggest that variations in an item's processing during the encoding stage may elicit different amounts of contextual cues for later retrieval, thus promoting differences in recollection-based retrieval. In contrast, similar variations in processing during retrieval may not produce helpful contextual cues, hence should not promote an effect on recollection-based retrieval.

Support for the notion that memory stage can predict the contribution to recollection can be found in [41]. These researchers manipulated the amount of attention allotted to the study and test stages. Their findings indicated that estimates of recollection were more susceptible to an attentional manipulation at encoding than at retrieval. In contrast, familiarity was affected at both encoding and retrieval by the manipulation.

In three experiments, we used identical manipulations, materials and procedures at encoding and retrieval to test our hypothesis. In Experiment 1, we tested the conceptual fluency manipulation [9], in which target words are presented in predictive as compared to not predictive sentences. The fluency manipulation has typically been used at retrieval without affecting recollection. We asked whether when manipulated at encoding, this manipulation would now affect recollection. Then, in Experiments 2 and 3, we asked the reverse question, whether a manipulation typically shown to affect recollection when carried out at encoding will not do so when manipulated at retrieval. Experiment 2 was designed to replicate—using the Experiment-1 materials—a finding by Knott and Dewhurst, who showed an effect of divided-attention for recollection when attention was manipulated at encoding but not when manipulated at retrieval [40] [41] [42]. In Experiment 3, we attempted to extend this finding using a different attentional manipulation and a different set of participants and materials (i.e., faces).

## Experiment 1

In Experiment 1, we used a fluency manipulation [8] [9]. This manipulation was originally designed to affect familiarity when carried at retrieval. We explored whether, when carried out at encoding, this very same manipulation would affect recollection. Participants were presented with sentences that either predicted terminal target words by constraining the possible candidates—the fluent condition—or did not predict target words—the non-fluent condition [8] [9]. For example, the target word “BOAT”, is predictable to a greater extent when it is embedded in the sentence “The stormy sea tossed the _____” (i.e., the fluent condition), than when embedded in the sentence “She saved her money and bought a _____” (i.e., the non-fluent condition). We assumed that target words in the fluent condition would be processed faster than target words in the non-fluent condition and would, therefore, be perceived as more familiar.

To date, this fluency manipulation has been carried out to examine overall recognition (but see [22]) not only at retrieval [9] but also at encoding [43]. However, the effects of this manipulation on recollection and familiarity have never been examined at encoding. Therefore, the results of our experiment may inform us not only regarding the effects of the fluency manipulation on recollection and familiarity at retrieval [22], but also on its contribution to recollection and familiarity when performed at encoding as compared to retrieval.

Because judgments based on fluency are considered to be automatic, they should influence familiarity, which is considered an automatic, heuristic process. Therefore, we predicted that when manipulated at retrieval, a fluency effect would be found for familiarity but not for recollection (cf. [21]). Critically, because recollection entails retrieval of contextual information from the encoding stage, when manipulated at encoding, an effect of fluency should be found for recollection.

### Method

#### Participants

Forty-eight Tel-Aviv University students (mean age = 22.56 years, SD = 1.31 years) participated in the experiment in exchange for course credit. This study was approved by the ethics committee for studies in psychology at Tel-Aviv University. Also, all participants gave their consent to participate in the experiments by pressing a key when they were prompt to declare that they agree to participate. They were informed that they can terminate their participation at anytime by pressing the "escape" key on the keyboard. Participants' consent was recorded in the data file of each experiment.

#### Design, Apparatus and Materials

Fluency of processing (fluent, non-fluent) was manipulated within subject in two separate blocks, with block-order counterbalanced across participants. The two blocks were the encoding-manipulation block (with fluency manipulated at encoding) and the retrieval-manipulation block (with fluency manipulated at retrieval).

In all, 120 Hebrew nouns served as target stimuli. Following Whittlsea [9], each target word was chosen so as to be the terminal word of each two types of sentences—fluent and non-fluent. Thus, a total of 240 sentences were created. Results of a pilot study of 10 participants confirmed that in the fluent sentences, the target word could be anticipated with a high probability, whereas in the non-fluent sentences, the target word was unlikely to be anticipated. Also, reading latencies of the fluent (*M* = 2440 ms, *SD* = 236.65) and non-fluent (*M* = 2366 ms, *SD* = 389.48) sentences were found to be equal (*t*(9) = 0.795, *p* = .447). Four additional words served as buffers and corresponded to only one of the two types of sentence. Target words were presented in gray Arial font (bold), size 60.

In the encoding-manipulation block, the study list comprised 30 words, corresponding to 15 fluent and 15 non-fluent sentences. At test, 60 stand-alone words (i.e., not assigned to any sentences), half old and half new, were presented. In the retrieval-manipulation block, the study list comprised 30 stand-alone words. At test, 60 words, half studied and half unstudied, equally distributed between the fluent and non-fluent conditions, were presented as terminal words within sentences. All conditions were counterbalanced across participants, such that each word appeared an equal number of times as old and new, and an equal number of times in the fluent and non-fluent conditions. In addition, in each block, items—single-words or sentences—were presented randomly.

#### Procedure

Participants were first introduced to the general structure of the experiment and with the characteristics of remember, know and guess (RKG) responses, as described by Gardiner et al. [16]. Specifically, following Gardiner et al., participants were also informed that they could give a 'guess' response, if the judgment of the item as ‘old’ could be attributed by the participant to neither recollection nor familiarity. After a short practice session, participants were prompted to declare whether they agree or not agree to participate in the study.

In the encoding-manipulation block, each study trial began with a '+' sign presented for 300 milliseconds, followed by a blank screen for 150 milliseconds. The fluent and non-fluent sentence then appeared on the screen, and participants were to first read the sentence, then to press the space bar on a computer keyboard. Only then–following a 250ms interval–the target word appeared in the terminal position. The terminal word remained on the screen for 1000 milliseconds, during which participants were to read it out loud and study it for the subsequent memory test.

Also in the retrieval-manipulation block, each study trial began with a '+' sign presented for 300 milliseconds, followed by a blank screen for 150 milliseconds. Then, the to-be-remembered words appeared at study without the preceding sentence. In both blocks, the offset of the target word was followed by a blank screen for 150 milliseconds.

To increase the effect of the fluency manipulation on familiarity (cf. [44] [45]), a distractor task preceded the recognition test in both the encoding- and the retrieval-manipulation blocks. In the distractor task, participants classified tones to “Low”, “Medium” and “High” for two minutes, by pressing designated keys on a computer keyboard.

At test, in both the encoding-manipulation block and the retrieval-manipulation block, target words were presented until a response was made. Participants read the target words out loud, judged whether the word was old or new, and then, if an 'old' judgment was made, performed a RKG judgment. After the judgments were typed in by the participants, a blank screen was presented for 200 milliseconds, followed by the next test trial. The encoding-manipulation and the retrieval-manipulation blocks were identical, with the exception that only in the retrieval-manipulation block, target words were preceded by a fluent or non-fluent sentence. When a sentence was presented, participants read it out loud, pressed the space bar, and then, after an interval of 250 ms, the target word appeared as terminal word in the sentence.

### Results and Discussion

We present the data corrected for the estimates of recollection and familiarity: We first calculated, for each participant, the proportion of Remember (R) and Know (K) responses by dividing a given response by the total number of non-guess responses, that is, by the total number of responses excluding guess responses. To illustrate, given that each experimental condition contained 20 items, the R proportion was calculated as: $P\left(R\right)\;=\frac{R}{20-G}\text{P(R) =}$ where R and G are the raw number of R and G responses. The subtraction of G responses from the total number of trials was performed after post-test debriefing revealed that G responses were mainly due to experimental noise. For example, participants typically responded with G when they pressed the "old" button by mistake, or when they could not decide when an item was old or new. Overall G rates were very small (*M* = .016, *SD* = .035 in the encoding-manipulation block; *M* = .038, *SD* = .071 in the retrieval-manipulation block), and were equally distributed over the experimental conditions (*t*(47) = 0.275, *p* = .785 in the encoding-manipulation block; *F*(3,141) = 0.828, *p* = .480 in the retrieval-manipulation block) (cf., [46]).

Thus, given that each experimental condition contained 20 items, Proportion Rs and Ks were calculated as:

$$P\left(R\right)\;=\frac{R}{20-G}\text{P}$$

$$P\left(K\right)\;=\frac{K}{20-G}\text{P}$$

where R, K and G are the raw frequencies of R, K and G responses, respectively.

Then, R and K proportions were applied to the correction for the estimates of recollection and familiarity according to Yonelinas and Jacoby's correction formulas [47] [48]. Recollection was estimated by the R proportion:

Recollection=P(R)Recollection

and familiarity was estimated by:

$$Familiarity=\frac{P(K)}{1-P(R)}\text{Familiarity}$$

The raw proportions of R and K responses are presented in S1 Table. Effect sizes throughout the article were calculated as Cohen's d.

To examine whether our manipulation replicated the well-documented fluency advantage for total recognition scores carried out at retrieval [9], we conducted two directional t-tests of dependent samples. Specifically, we compared recognition scores for items that were studied in the fluent condition, which were higher (mean = 0.80, SE = 0.02) than those studied under the non-fluent condition (mean = 0.75, SE = 0.02). This difference was significant, *t*(47) = 1.84, *p* < .05, *d* = 0.36. Also for unstudied items, recognition scores for items that were processed fluently (mean = 0.33, SE = 0.03) were higher than recognition scores for items that were not processed fluently (mean = 0.23, SE = 0.02). This difference, too, was significant, *t*(47) = 4.56, *p* < .01, *d* = 0.58. Therefore, the original fluency effect on overall recognition was replicated.

Next, we turned to examine the influence of the fluency manipulation, carried out at retrieval, on the estimates of recollection and familiarity. Separate analyses are reported for hits and for FA. Note that the influence of study stage could not be assessed for the FA data because when the fluency manipulation was manipulated at encoding, it could not—by definition—be applied to unstudied items. Thus, here and in Experiment 3, FAs are only reported in the retrieval-manipulation block, wherein the manipulation was undertaken for both unstudied and studied items. In contrast, for hits, performance could be reported as a function of the memory stage, because for studied items, the fluency manipulation was carried both in the retrieval-manipulation block and in the encoding-manipulation block.

Examination of the data revealed that for recollection, the FAs in the fluent condition (mean = 0.04, SE = 0.01) were only slightly higher than the FAs in the non-fluent condition (mean = 0.03, SE = 0.01). Due to their small size, these responses were not submitted to statistical analysis. As for familiarity, the FAs in the fluent condition (mean = 0.25, SE = 0.02) were higher than the FAs in the non-fluent condition (mean = 0.18, SE = 0.02). A dependent samples t-test revealed this increase in familiarity as a function of fluency to be significant, *t*(47) = 3.84, *p* < .01, *d* = 0.51. Thus, for FAs, when manipulated at retrieval, the fluency manipulation significantly affected familiarity.

For hit rates, the mean estimates of recollection and familiarity are presented in Table 1. Examination of the estimates revealed a fluency effect for familiarity when the manipulation was carried out at retrieval, but not at encoding. Most important, as predicted, the recollection data showed the opposite pattern. The fluency effect was found for recollection only when the manipulation was carried out at encoding, but not at retrieval.

**Table 1:** Experiment 1. Mean estimates (and SE) of Recollection and Familiarity hit rates, as a function of Fluency (Fluent, Non-fluent) and Memory stage (Encoding, Retrieval).

		Fluency		
Process	Memory stage	Fluent	Non-Fluent	Cohen's *d* ^a^
Recollection	Encoding	.65 *(*.*03)*	.58 *(*.*03)*	0.34
	Retrieval	.39 *(*.*03)*	.42 *(*.*03)*	-0.14
Familiarity	Encoding	.54 *(*.*05)*	.58 *(*.*04)*	-0.13
	Retrieval	.60 *(*.*03)*	.51 *(*.*03)*	0.43

Note. Recollection and Familiarity were estimated using Yonelinas and Jacoby's correction formulas [47].

^a^ Cohen's d represents the effect size of the fluency manipulation (for details and interpretation, see [75]).

We submitted each of the interactions to an ANOVA, with both memory stage (Encoding, Retrieval) and fluency (Fluent, Non-fluent) manipulated within subject. For familiarity, the fluency X memory stage interaction was significant, *F*(1, 47) = 5.52, *MSE* = 0.04, *p* < .05. At encoding, no significant effect of fluency on familiarity was found, *F* < 1. In contrast, at retrieval an effect was found, with higher estimates of familiarity under the fluent condition than under the non-fluent condition, *F*(1, 47) = 5.70, *MSE* = 0.03, *p* < .01.

For recollection too, the fluency X memory stage interaction was significant, *F*(1, 47) = 9.47, *MSE* = 0.01, *p* < .01. Critically, however, this interaction was opposite in direction to the one found for familiarity. As predicted, the source of this interaction was an increase in estimated of recollection in the fluent, as compared to the non-fluent, condition, but only at encoding, *F*(1, 47) = 10.98, *MSE* = 0.01, *p* < .01. At retrieval, however, no significant effect of fluency of recollection was found, *F*(1, 47) = 2.14, *MSE* = 0.01, *p* > .05.

Two aspects of the results are noteworthy. First, Whittlesea's fluency manipulation was found to affect familiarity hits when carried out at retrieval, with no effect found on recollection. Though Whittlesea [9] did not examine the separate influence of the fluency manipulation to recollection and familiarity, the theoretical analysis that he ascribed to this manipulation corresponds to the finding of an influence on familiarity but not recollection (cf., [22]).

Second, and of primary interest to our present concerns, the results of this experiment provide evidence for our notion that recollection is dependent on the processing that information undergoes at encoding. Thus, at encoding, fluency affected recollection but not familiarity, with higher recollection in the fluent condition. These results suggest that sensitivity to fluency is not unique to familiarity. Rather, the answer to the question of whether fluency affects recollection or familiarity depends on the memory stage at which the manipulation is carried out. When carried out at retrieval, fluency indeed affects familiarity. Critically, however, when carried out at encoding, it affects recollection. This supports our thesis that the question of *how* the fluency manipulation affects recollection may be ill conceived and may be tangential to the critical element of the intimate relationship between recollection and encoding.

## Experiment 2

In Experiment 1, we demonstrated that fluency—a retrieval manipulation which has heretofore not been found to affect recollection—produced an effect on recollection when manipulated at encoding. We interpreted these findings as supporting our thesis that manipulations undertaken at encoding create variations in the context in which the information is stored. Therefore, because the process of recollection entails retrieving information that includes contextual detail, manipulations carried out at encoding—at the time when context is bound with the item—should affect recollection.

In contrast to this interpretation, one could argue that encoding is not the critical factor determining the presence of recollection, but rather, the characteristics of the manipulation is possibly affected when shifting from retrieval to encoding (cf., [13]). Specifically, when fluency was manipulated at retrieval, it affected processing speed or fluency-of-processing. However, when this manipulation was shifted from retrieval to encoding, it no longer affected processing speed or fluency-of-processing. Rather, it now affected the level of attention directed to stimuli (or the amount of distinctive information which was extracted). This change in the nature of the manipulation enabled recollection to emerge. Accordingly, the conclusions of our demonstrations may more correctly be interpreted in terms of the characteristics of the manipulations—which could be argued to change when moving from retrieval to encoding—rather than in terms of the memory stage during which they were carried out.

The argument against a memory-stage interpretation of our findings, targets the construct validity (e.g., [49]) of the experimental manipulation, suggesting that the experimental manipulation carried out at encoding does not represent the same theoretical variable as the exact same manipulation carried out at retrieval. In Experiment 2, we wish to provide a rebuttal to this argument by examining an additional manipulation, designed to affect attention. In particular, an attention-manipulation entails the exact same process—that of reducing or elevating the amount of available resources—whether carried out at encoding or at retrieval (though, possibly, because encoding and retrieval are different operations, they may still differ in their resource-dependency). Because the manipulation of attention is based on a well-defined theoretical structure, it would be more difficult to defend the idea that the characteristics of this manipulation varies as a function of the memory stage.

In a recent study, Knott and Dewhurst manipulated participants' attention both during encoding and retrieval [41]. These authors measured R and K responses for memory of recognized words. At encoding, Knott and Dewhurst replicated the typical (e.g., [7] [19]) pattern of divided attention (DA) at encoding, with R responses affected to a greater extent by divided attention than K responses [42]. However, at retrieval, DA affected K responses to a greater extent than it affected R responses [40] [41] [42]. Thus, DA has been demonstrated to produce different effect on R and K responses at encoding as compared to retrieval [41], in a manner analogous to that which was found in Experiment 1, using the fluency manipulation.

Critically, it is difficult to evaluate the effect of memory stage on R/F judgments across the two manipulations (fluency, DA), in that the two manipulations were confounded by different labs, materials, designs and procedures. Experiment 2 was thus designed to replicate Knott and Dewhurst's findings [41], using the same materials, design and procedure as those used in Experiment 1. We hypothesized that recollection-based retrieval of words will be reduced when attention is divided at encoding, but not at retrieval.

### Method

#### Participants

In exchange of course credit, 68 first-year psychology students at Tel-Aviv University participated in this study (mean age = 23.55, *SD* = 3.72; 50 females). The study was approved by the university ethics committee, with written consent of willingness to participate in the study, provided by participants.

#### Design, Materials, and Procedure

Attention (full, divided at encoding, divided at retrieval) was manipulated within subject, in three separate blocks. In the first block, participants performed the memory task with full attention at both encoding and retrieval. The two remaining blocks were the encoding-manipulation block—in which attention was divided only at encoding—and the retrieval-manipulation block—in which attention was divided only at retrieval). Presentation order of the encoding- and the retrieval-manipulation blocks was counterbalanced across participants.

Memory was assessed using the same materials as in Experiment 1. Specifically, 120 Hebrew nouns served as target stimuli and were randomly assigned to three blocks of 40 words. Six additional words served as buffers, with two words presented at the beginning of each of the three test blocks. All words were presented in black Arial font (bold), size 60.

In each of the blocks, the study list comprised 20 words, presented randomly at a rate of 1000 ms per word. Each word was preceded with a '+' sign presented for 250 ms, followed by a blank screen for 100 ms. The offset of the target word was followed by a blank screen for 100 ms. At test, 40 words were presented randomly, of which half were studied and half were new. Participants were asked to determine for each test word whether it was studied or not studied, by pressing designated buttons on a computer keyboard, marked with red (the L key; "not studied") and blue (the A key; "studied") stickers. If a word was judged as studied, participants were prompted to perform a RKG judgment, as in described Experiment 1. The test was self-paced. The three 40-words sets were counterbalanced across the three attention conditions, such that across participants, each set appeared an equal number of times under the full, divided-at-encoding, and divided-at-retrieval conditions.

Attention was divided using a secondary task of tone classification [7]. This task comprised three tones of 220Hz (low), 440Hz (medium), and 880Hz (high). The tones were generated by professional audio software, and were 550 ms long. In the secondary task, participants were to classify tones as “Low”, “Medium”, or “High”, by pressing designated keys on a numeric keypad connected to a computer. The tones were presented randomly, with a 300ms inter-stimulus interval (ISI). If a given tone was not classified within the 850 ms interval (tone on-set plus ISI), a new tone was presented.

In each of the three blocks, participants performed the tone-classification task for 30 seconds between the study and test stages, thus serving as a distractor task. More importantly, the task was performed during the presentation of the study list in the encoding-manipulation block, and during the recognition test in the retrieval-manipulation block. Participants were informed that accuracy was very important for both the memory and the tone-classification tasks, and that they should perform as fast as they could in the tone-classification task.

Participants were given detailed instructions of the RKG recognition task and of the tone-classification task at the beginning of the experiment. In addition, each block began with a reminder of the instruction for the forthcoming task, as well with a short practice that simulated the forthcoming task in that block.

### Results and Discussion

As a manipulation check, we first turned to examine the effect of the attention manipulation on overall recognition scores. We first observed that overall recognition was highest under full attention (mean = 0.88, SE = 0.02), lower for DA-at-retrieval (mean = 0.76, SE = 0.02), and was lowest for the DA-at-encoding condition (mean = 0.64, SE = 0.02). We applied the data to a one-way repeated-measures analysis of variance (ANOVA), and found significant differences, *F*(2,134) = 75.04, *p* < .001. A series of three dependent samples t-tests was employed as a post-hoc analysis, with the significance level reduced to .017, according to Bonferroni's correction for multiple comparisons. The analyses revealed that when attention was divided at encoding, recognition scores for studied items were lower as compared to performance under full attention, *t*(67) = 12.79, *p* < .001, *d* = 3.13. When attention was divided at retrieval, recognition scores for studied items were all lower as compared to performance under full attention, *t*(67) = 6.58, *p* < .001, *d* = 1.61. Finally, when attention was divided at retrieval, recognition scores for studied items were higher than when attention was divided at encoding, *t*(67) = 5.49, *p* < .001, *d* = 1.34.

As for unstudied items, a one-way repeated-measures analysis of variance (ANOVA) found significant differences between the three attention conditions, *F*(2,134) = 11.35, *p* < .001. A series of dependent samples t-tests revealed that when attention was divided at encoding, false-alarm rate was higher (mean = 0.27, SE = 0.02) as compared to false-alarms under full attention (mean = 0.21, SE = 0.02), *t*(67) = 2.66, *p* = .01, *d* = 0.65. When attention was divided at retrieval, false-alarm rates were lower (mean = 0.16, SE = 0.02) as compared to false-alarms under full attention, *t*(67) = 2.01, *p* = .05, *d* = 2.01. Finally, when attention was divided at retrieval, false-alarm rates were lower than when attention was divided at encoding, *t*(67) = 5.04, *p* < .001, *d* = 1.23. The effects of the attentional manipulation on total recognition scores replicates the asymmetry between encoding and retrieval processes reported in the literature (e.g., [50]), such that manipulating attention at encoding hurt memory performance for studied items more so than did manipulating attention at retrieval.

Next, we turned to examine the effects of the attention manipulation on recollection and familiarity. As in Experiments 1, we present the data corrected for the estimates of recollection and familiarity. Mean estimates of Recollection and Familiarity hits are presented in Table 2. Raw proportions of R and K responses are presented in S2 Table.

**Table 2:** Experiment 2. Mean estimates (and SE) of Recollection and Familiarity hit rates, as a function of Attentional: Full, divided at encoding, and divided at retrieval.

	Attention manipulation		
Process	Full	Divided at encoding	Divided at retrieval
Recollection	.58 *(*.*03)*	.22 *(*.*02)*	.51 *(*.*03)*
Familiarity	.67 *(*.*04)*	.49 *(*.*02)*	.42 *(*.*04)*

Note. Recollection and Familiarity were estimated using Yonelinas and Jacoby's correction formulas [47].

For recollection, a one-way repeated measures ANOVA revealed a significant difference between the three attention conditions, *F*(2,134) = 67.02, *p* < .001. Three dependent-samples t-tests (with significance level of .017) revealed that recollection hits were lower when attention was divided at encoding, as compared to full attention, *t*(67) = 10.22, *p* < .001, *d* = 2.50. Recollection hits were slightly lower when attention was divided at retrieval as compared to full attention, yet this difference did not reach significance, *t*(67) = 2.37, *p* = .02, *d* = 0.58. Finally, recollection hits were lower when attention was divided at encoding, than when it was divided at retrieval, *t*(67) = 8.37, *p* < .001, *d* = 2.05.

For familiarity, a one-way repeated measures ANOVA revealed a significant difference between the three attention conditions, *F*(2,134) = 21.76, *p* < .001. Three dependent-samples t-tests (with significance level of .017) revealed that familiarity hits were lower when attention was divided at encoding, as compared to full attention, *t*(67) = 5.51, *p* < .001, *d* = 1.35. Familiarity hits were also lower when attention was divided at retrieval, as compared to full attention, *t*(67) = 5.33, *p* < .001, *d* = 1.30. Finally, there was no difference between familiarity hits when attention was divided at encoding, as compared to divided at retrieval, *t*(67) = 1.76, *p* = .08, *d* = 0.43.

Next, we examined the effect of the attention manipulation on familiarity false-alarms (FAs). As in Experiment 1, we do not report or analyze recollection FAs due to their small sizes (less than 3%). A one-way repeated measures ANOVA revealed that familiarity FAs were affected by the attention manipulation, *F*(1,134) = 25.20, *p* < .001. Thus, FAs when attention was divided at encoding (mean = 0.17, SE = 0.02) were higher than under full attention (mean = 0.06, SE = 0.01), *t*(67) = 6.99, *p* < .001, *d* = 1.71. FAs when attention was divided at retrieval (mean = 0.09, SE = 0.01) did not differ from FAs under full attention, *t*(67) = 1.53, *p* = .13, *d* = 0.37. Finally, FAs when attention was divided at encoding were higher than when attention was divided at retrieval, *t*(67) = 4.87, *p* < .001, *d* = 1.19.

Taken together, the findings presented here support our notion that recollection will be more affected by manipulations carried at encoding, but not when carried at retrieval. In this experiment, recollection-based retrieval was dramatically decreased when attention was divided at encoding, yet was not affected when attention was divided at retrieval. While comparable evidence was already presented in Experiments 1, here it is more difficult to provide an alternate interpretation of the results in terms of differences in underlying theoretical constructs.

Put differently, our findings indicate an asymmetry between encoding and retrieval in regard to the effects of divided-attention manipulation on memory performance. Such an asymmetry is well documented in free-recall and in total recognition scores (e.g., [50]). These researches suggested that although encoding and retrieval are attention-demanding processes, retrieval is more resilient to divided attention.

Other researchers too ([40] [51]) have reported that recollection, but not familiarity, is sensitive to changes in attention during encoding but not to changes during retrieval. Importantly, however, their findings were not interpreted, as we propose here, in terms of the different effects of memory stage. To reiterate, we wish to argue that manipulations undertaken at encoding create variations in the context in which the information is stored. Therefore, because the process of recollection entails retrieving information that includes contextual detail, only manipulations carried out at encoding—at the time when context is bound with the item—should affect recollection.

Rather than interpreting the different influence of encoding and retrieval on R in terms of memory stage, Knott and Dewhurst ([40]; see also [51]) interpreted this difference in terms of Rajaram's fluency-distinctiveness model [13]. This fluency-distinctiveness model suggests that changes in attention during encoding alter item's distinctiveness, thus affecting recollection. Changes in attention during retrieval may not alter item's distinctiveness, thus recollection was not affected.

Critically, Rajaram's fluency-distinctiveness model can just as easily apply to the findings presented in our Experiment 2. In particular, one could argue that the attention-demanding tone-classification task interfered with participants' ability to encode study words in a distinctive manner, thus only a few of these words "achieved" distinctiveness. This manifested in low recollection rate. We address this possible explanation in Experiment 3.

## Experiment 3

Thus far, we have demonstrated that recollection is more likely to be affected by manipulations carried at encoding, rather than manipulations carried at retrieval. However, a leading account of the processes underlying R judgments is that it is the analysis of the distinctive or salient attributes of the information creates memories that are later accompanied by Remember responses [13]. Therefore, we next wished to generalize our findings to stimuli which were highly distinctive, unlike the word-stimuli used in Experiment 2. Specifically, if R responses are indeed influenced by increased processing of distinctive information at encoding, as suggested by Rajaram, then using stimuli which are already highly distinctive, should eliminate, or at least mitigate, any possible effect on R responses. If, however, R responses are mediated by processing of information at encoding per se, then the distinctive nature of the stimuli should not matter, and an influence on R response should be observed for distinctive stimuli, as it was observed for word stimuli.

To this end, in Experiment 3, we examined unfamiliar faces, which comprise a class of stimuli which are highly distinctive. Indeed, we chose facial stimuli which had particularly distinctive features, such as a long nose or crooked eyes. If an effect would be observed even for these unique stimuli, then the notion that an analysis of distinct features is insufficient to account for RK performance would be bolstered.

Consistent with our hypothesis, we predicted that at encoding, the attentional manipulation would affect recollection but that recollection should not be affected by the DA manipulation during retrieval. Thus, an interaction was expected between the stage in which the manipulation is carried-out (Encoding, Retrieval), and the amount of attentional-load (Low-load, High-load).

### Method

#### Participants

Twenty-four Tel-Aviv University students (mean age = 23.47 years, SD = 2.15 years) participated in the experiment in exchange for course credit. This study was approved by the ethics committee for studies in psychology at Tel-Aviv University. Also, all participants gave their consent to participate in the experiments by pressing a key when they were prompt to declare that they agree to participate. They were informed that they can terminate their participation at anytime by pressing the "escape" key on the keyboard. Participants' consent was recorded in the data file of each experiment.

#### Design

Attentional-load (Low-load, High-load) was manipulated within subject in two separate blocks—which were also manipulated within subject. The two blocks—corresponding to memory stage—were the encoding-manipulation and the retrieval-manipulation blocks, with attentional load manipulated at encoding and retrieval, respectively.

#### Apparatus and Materials

A total of 128 photos of human faces were used in this experiment. Photos were taken from the Karolinska Directed Emotional Faces database (KDEF; [52]) and downloaded from the Internet, with the constraint that each photo should bare a unique—exaggerated—extreme facial expression, hairstyle, color, gender, age, and position in the picture frame. All photos were of high quality and standardized to 225 pixels width and 305 pixels height.

The experiment consisted of two experimental blocks. In the encoding-manipulation block, attentional load was manipulated at study (as described below), with 15 photos presented in the high-load and 15 photos in the low-load condition. At test, 30 old and 30 new photos were presented without an attentional manipulation. In the retrieval-manipulation block, at study, participants studied 30 photos, without an attentional manipulation. At test, 30 old and 30 new photos were presented with each type equally divided between the high-load and the low-load conditions.

Because pictures are spatial stimuli, attention was not manipulated using the tone classification task (a spatial task), but rather with a verbal task. Specifically, to manipulate attention, each photo was randomly paired with either a common 6-letter Hebrew word, taken from a contemporary Hebrew-Hebrew dictionary (e.g., SCANNER) or with a 6-letter string of an identical letter (e.g., AAAAAA). Across trials, each word was paired an equal number of times with a word and with an identical-letter string. To manipulate attention, the 6-letter string or word was presented and participants had to recite the letters backwards. After 750 ms., the word or letter-string were replaced by the photo for 2000 ms. During the presentation of the photo, participants were required to continue reciting the letters from back to front from memory. For identical-letter strings, this was a relatively easy task—constituting the low-load condition. However, for words, this entailed maintaining the word in memory and constantly retrieving its spelling—thereby constituting the high-load condition. All words and identical-letter strings were presented in black Tahoma font, size 40 and only appeared when attention was manipulated.

Eight photos served as buffers, two photos in each study or test stage. In addition, 10 extra photos and 10 extra words and identical-letter strings were used for a short practice.

#### Procedure

As in Experiments 1 and 2, individually tested participants were given printed instructions for the experiment, in which they were acquainted with the two experimental blocks and with the RKG procedure.

In a short practice session, participants were acquainted with the memory task and with the high- and low-load tasks. They were also encouraged to deeply encode the photos by generating, for each photo, a corresponding association. Following the practice session, participants were prompted to declare whether they agree or not agree to participate in the study, with the experiment then beginning.

Each block consisted of the presentation of 30 photos. Each trial started with a '+' sign presented at the middle of the screen for 1000 ms., followed by a blank screen for 500 ms. Subsequently, the target photo was presented for 2000 ms., after which it was masked with a black rectangle for 250 ms. As described above, for the attention manipulation (at either study or test)—prior to the presentation of the target photo—a word (high-load condition) or the letter-string (low-load condition) was presented for 750 ms, and was replaced by the photo.

At test, a message appeared on the screen informing participants about the upcoming recognition test. As described above, test trials had the same structure as study trials, except the offset of each target photo was followed by an “old/new” judgment notification on the screen. Participants performed the judgment by pressing a designated key on the keyboard for the different judgments. When the word was judged as old, an RKG judgment notification appeared on the bottom of the screen, and participants pressed the designated keys according to their judgment. Immediately after making the RKG judgment, a new trial began.

### Results and Discussion

As in Experiments 1 and 2, we present the data, corrected for the estimates of recollection and familiarity. Raw proportions of R and K responses are presented in S3 Table.

As a manipulation check, we first turned to examine the effect of the attentional-load manipulation on overall recognition scores. When the manipulation was carried out at encoding, recognition scores for items that were studied under low-load (mean = 0.84, SE = 0.03) were higher than those under high-load (mean = 0.57, SE = 0.04). This difference was significant, *t*(23) = 8.86, *p* < .01, *d* = 1.57. When the manipulation was carried out at retrieval, recognition scores for studied items that were retrieved under low-load (mean = 0.91, SE = 0.02) were slightly higher than those retrieved under high-load (mean = 0.88, SE = 0.03). This difference was not significant, *t*(23) = 1.20, *p* > .05, *d* = 0.24. As for unstudied items, false alarms for items that were retrieved under low-load (mean = 0.10, SE = 0.02) were lower than those retrieved under high-load (mean = 0.21, SE = 0.03). This difference was significant, *t*(23) = -5.00, *p* < .01, *d* = -0.9. The effects of the attentional-load manipulation on total recognition scores replicated the asymmetry between encoding and retrieval processes found in Experiment 2.

Next we asked whether a similar asymmetry can be found on the effects of attention on the estimates of recollection of studied items. Mean estimates of Recollection and Familiarity hits are presented in Table 3.

**Table 3:** Experiment 3. Mean estimates (and SE) of Recollection and Familiarity hit rates, as a function of Attentional-load (High, Low) and Memory stage (Encoding, Retrieval).

		Attentional-load		
Process	Memory stage	Low	High	Cohen's *d* ^a^
Recollection	Encoding	.43 *(*.*05)*	.21 *(*.*03)*	1.12
	Retrieval	.53 *(*.*05)*	.49 *(*.*06)*	0.15
Familiarity	Encoding	.68 *(*.*05)*	.46 *(*.*04)*	1.00
	Retrieval	.77 *(*.*06)*	.75 *(*.*05)*	0.07

Note. Recollection and Familiarity were estimated using Yonelinas and Jacoby's correction formulas [47].

^a^ Cohen's d represents the effect size of the attentional-load manipulation (for details and interpretation, see [75]).

Examination of Table 3 revealed two interactions. First—as predicted—for recollection, an attentional-load effect was found when the manipulation was carried out at encoding, but not at retrieval. Second, for familiarity, a similar interaction was found, with attention affecting familiarity when the manipulation was carried out at encoding and less so, when at retrieval.

We submitted each of the interactions separately to an analysis of variance (ANOVA), with both memory stage (Encoding, Retrieval) and attentional-load (Low, High) as within-subject variables. For recollection, the attentional-load X memory stage interaction was significant, *F*(1, 23) = 13.97, *MSE* = 0.02, *p* < .01. The simple effects revealed that at encoding, the higher attentional load decreased recollection, *F*(1, 23) = 43.40, *MSE* = 0.01, *p* < .01, while at retrieval it did not, *F*(1, 23) = 1.40, *MSE* = 0.01, *p* > .05.

For familiarity, examination of the estimates revealed that the attentional-load X memory stage interaction was also significant, *F*(1, 23) = 5.81, *MSE* = 0.04, *p* < .05. At encoding, the simple effects revealed that high attentional-load decreased familiarity, *F*(1, 23) = 22.27, *MSE* = 0.02, *p* < .01, while at retrieval there was no effect on familiarity, *F*(1, 23) < 1.

Examination of the estimates of the false-alarms (FA) revealed that the attentional-load manipulation affected familiarity. As in Experiments 1 and 2, these data can only be reported for the retrieval-manipulation block, in which the manipulation was undertaken for unstudied as well as for studied words. For familiarity, the FAs in the low-load condition (mean = 0.08, SE = 0.02) were lower than the FAs in the high-load condition (mean = 0.15, SE = 0.03). This effect was significant, *F*(1, 23) = 21.12, *MSE* = 0.003, *p* < .01.

The results of Experiment 3 lend further support to our notion that the stage in which a manipulation is carried out—rather than the type of manipulation per se’—best determines whether recollective processes would be affected. Specifically, when employed at retrieval, the attentional-load manipulation did not affect recollection or familiarity hits. Critically, in accordance with our prediction, when employed at encoding, this same manipulation did affect recollection.

Opposed to Rajaram's fluency-distinctiveness model [13], our study eliminated the ability to rely on item's distinctiveness during retrieval, because all items, both old and new, were highly distinctive. Thus, the fact that recollection was affected by changes in attention during encoding but not during retrieval could not be easily attributed to changes in item's distinctiveness, but rather to the stage in which the manipulation was carried. Hence, recollection was more likely to be affected when variations in processing took place at encoding, than at retrieval.

Still, a few studies have reported an affect of divided attention on recollection at retrieval [53] [54]. Note, however, that in these studies, the effects of DA at retrieval on recollection were obtained by manipulating attention along with several other manipulations within a single design [53]. The inclusion of several variables makes it difficult, if not impossible, to isolate the effects of the DA manipulation.

A different difficulty in interpretation is found in a study by Gruppuso et al. [54]. In their study, attention was divided using a secondary task which had similar semantic structure to the primary-memory task. Therefore, in their study, the effect of DA at retrieval on task performance may have stemmed from the competition for semantic-resources rather than from a competition for attentional-resources.

In contrast, in our study, we intentionally used primary and secondary tasks from separate domains, face processing and word reading, respectively. Because both the primary task and the secondary task—reading the word or the identical-letter string—relied on memory, both tasks competed directly for attentional-resources required for memory performance. By doing so, we replicated the finding of a decrease in recollection as a function of DA at encoding. More important, we were able to demonstrate that recollection is not affected by the same manipulation carried out at retrieval as when it is carried out at encoding.

When manipulated at encoding, our manipulation also affected familiarity. Although this effect was not predicted, it did not contradict any of our predictions. This effect was probably due to non-criterial recollection, which is a form of recollection that is manifested in the estimates of familiarity. In the General Discussion we describe the idea of non-criterial recollection and discuss possible reasons for its appearance in this experiment and in Experiment 2, but not in Experiments 1.

## General Discussion

Our goal was to investigate whether the memory stage (encoding, retrieval) in which a manipulation is carried out can help predict whether recollection is affected. Our results suggest that when the manipulation is carried out at encoding, recollection is likely to be affected. In contrast, when the manipulation is carried out at retrieval, recollection is likely to not be affected. Therefore, an encoding-retrieval asymmetry was found for the effects of manipulations on recollection.

In Experiment 1, we demonstrated that a manipulation designed specifically to affect familiarity—the fluency manipulation—affected recollection when carried out at encoding while not affect recollection—but affecting familiarity—when carried out at retrieval. In Experiments 2 and 3, we demonstrated that a manipulation known to affect recollection—the divided attention (DA) manipulation—indeed affected recollection (as well as familiarity; see discussion below on non-criterial recollection), but only when carried out at encoding. When attention was divided at retrieval, neither recollection nor familiarity was affected.

Put altogether, these findings suggest that the characteristics of recollection are better understood when considering the stage in which the manipulation is carried out. This finding is unintuitive in that both recollection and familiarity are retrieval processes and, as such, might both be expected to recover information encoded at study. However, our findings suggest that because recollection is a contextual-based retrieval process, it—but not necessarily familiarity—is influenced almost exclusively by the manner in which information was processed at encoding.

One caveat to this suggestion is that like recollection, familiarity too may at times be sensitive to manipulations carried out at encoding. Indeed, in Experiment 3, familiarity was also affected by the attentional-load manipulation that was carried out at encoding. Interestingly, though this pattern was revealed in the corrected scores, it was not found in the raw K responses reported in S3 Table. Either way, to the extent that an effect on familiarity was found from an encoding manipulation, it seems to devizate from our notion that only recollection is a context-based process, and therefore it alone should be influenced by study manipulations.

Still, this finding does not challenge our thesis [19] [41] [55]. Our thesis only focuses on the intimate relationship between encoding and recollection. The finding that encoding manipulations can affect familiarity has been interpreted as non-criterial recollection [56]. Non-criterial recollection was suggested to be the manifestation of recollection in estimates of familiarity, under conditions where retrieval is mainly recollection-based [57] [58]. Thus, the most accurate prediction we can make is that to the extent that an encoding manipulation affects recognition performance, it should always influence recollection and may at times also affect familiarity in the same direction as it affected recollection. In those cases, the effects of the manipulation on familiarity would be the product of non-criterial recollection.

Unfortunately, current understanding of non-criterial recollection is still limited and the conditions under which it appears can only be explained on a post-hoc manner. Though not predicted by us, the effects of divided attention at encoding on familiarity in Experiments 2 and 3, may suggest that perceptual distinctiveness may mediate the appearance of non-criterial recollection. According to this suggestion, in Experiment 3, not only standard facial-features, but also expression, hairstyle and color were encoded as contextual information. This makes the attribute of “uniqueness”, one which is not diagnostic with regard to its appearance in the study phase. Instead, only the more standard facial features or the retrieval of a combination of several contextual cues could fuel the process of recollection. We speculate that under such circumstances—where an abundance of non-diagnostic contextual-information is available—an “indirect” influence of the contextual information can affect familiarity in the guise of non-criterial recollection. Nevertheless, this explanation is well accommodated with the effects found on familiarity in Experiment 3, but not in Experiment 2. Yet, one should note that non-criterial recollection is typically observed for verbal stimuli [57] [59]; thus, it is possible that in Experiment 2 as well, the effect of attention on familiarity may be a manifestation of non-diagnostic contextual-information.

A second caveat to our thesis regarding the importance of the stage in which the manipulation is carried out, is that this suggestion can only apply to a limited—albeit large—set of manipulations that involve only a single memory stage. In this article, we demonstrated that fluency and attentional-load manipulations had a differential effect on recollection as a function of the stage in which the manipulation was carried out. Nevertheless, there are many manipulations for which we do not make any predictions, in that there is no clear stage at which they can be said to be carried out. Such manipulations include aging (e.g., [50]), amnesia (e.g., [34]), delay between encoding and retrieval (e.g., [60]) and word frequency (e.g., [61]). More research is needed to better understand when these manipulations influence recollection, when they influence familiarity and when they influence both (cf., [35]). Likewise, some manipulations (e.g., massed versus spaced presentation, e.g., [62]) can only be carried out at encoding, and so our thesis cannot be empirically tested for these manipulations (though, as predicted by our suggestion, they do show a consistent effect on recollection).

A final, third caveat has to do with an effect on recollection found when response-deadline is manipulated, in which the time allotted to retrieval is limited to less than 1000 milliseconds (i.e., the "fast" condition in [63]) or to more than 1000 milliseconds (i.e., the "slow" condition). The response-deadline manipulation can only be carried out at retrieval, and has typically been found to affect recollection.

We argue that our framework addresses manipulations that modulate the magnitude of recollection-based retrieval, reflected by different levels of recollection-based retrieval in performance. The response deadline procedure, in contrast, is designed to completely eliminate recollection-based retrieval—which it succeeds in doing. Specifically, recollection-based retrieval is typically initiated between 500–800 milliseconds after stimulus onset, whereas familiarity-based retrieval is typically initiated between 300–500 milliseconds (e.g., [64]). Thus, in the fast response-deadline condition, where response time is usually limited to 700 milliseconds (e.g., [65]) recollection-based retrieval has little chance of being initiated, and indeed, does not show up. Therefore, although this manipulation is carried out at retrieval, it affects recollection by eliminating it (in the fast condition) rather than by modulating its contribution to performance.

After delineating the limits of our thesis, we would like to focus of the big picture. The notion that the stage in which the manipulation is carried out predicts the processes to be affected can explain the majority of the data reported in the literature. For example, modality change affects recollection only when carried out at encoding but not at retrieval [66]. Likewise, levels of processing (e.g., [15]) and study duration (e.g., [62]) mainly affect recollection because these manipulations, too, are manipulated at encoding. Finally, semantic priming does not affect recollection (e.g., [21]) when manipulated at retrieval.

In contrast to our thesis, one could argue that there are several studies that demonstrated an effect on recollection by manipulations carried out at retrieval [22] [67] [68] [69]. However, most of these studies either did not estimate the independent contribution of recollection and familiarity (e.g., [68]) or did so by measuring changes in response-confidence levels (e.g., [22] [67]). Therefore, further work is needed in order to examine our notion that recollection is intimately related to the encoding stage, using different estimation methods of recollection and familiarity [70] [71] [72].

Critically, we do not rule out the notion that recollection may be affected by manipulations carried at retrieval; we argue that recollection is prone to be affected when some variability in the processing of to-be-remembered information can be attributed to the encoding stage (see also [54] [73]). A study also highly relevant to our current work is McCabe and Balota's study [69], in which medium-frequency target words were paired with low or high frequency unrelated prime words. When carried at encoding, high frequency primes increased estimates of recollection (but not of familiarity), as compared to low frequency primes. When carried at retrieval, this manipulation again increased estimates of recollection, but not of familiarity. McCabe and Balota explained their results within the expectancy-heuristic framework (see also [9]). According to this framework, participants expect an item to have a certain level of activation. If there is a discrepancy between the actual and the expected levels of activation, then this discrepancy is attributed to past occurrence of the item. Whereas Whittlesea [9] suggested that expectancy discrepancy is attributed to item's familiarity, McCabe and Balota [69] suggested that if an item reaches a certain amount of activation, expectancy discrepancy may be attributed to recollection. Therefore, recollection can be affected by manipulation carried at retrieval as well as at encoding. The findings presented in this article may be interpreted within the expectancy-discrepancy framework; we discuss it later on from a single process perspective.

An additional issue that needs to be addressed is the effect of encoding-retrieval compatibility on recollection and familiarity. Dewhurst and Brandt manipulated the depth to which information was processed (specifically, Read versus Anagram) at both encoding and retrieval [74]. These authors found that remember—but not know—responses were increased when information was presented in the same format (read, anagram) at encoding and retrieval (see also [65] and [13], for similar results using size congruency). However, in contrast to these findings, our findings suggest that encoding-retrieval compatibility cannot completely explain the contribution of recollection to memory performance.

Specifically, in our study, we deliberately manipulated the stage in which the manipulation was carried out in two separate blocks, thereby enabling us to examine the pure effects of the manipulation at encoding or at retrieval. For instance, in the encoding-manipulation block of Experiment 1, participants studied words in sentences that evoked a fluent or a non-fluent processing of the target word. At test, words were presented individually, without a sentence. Hence, there was no perceptual compatibility between encoding and retrieval presentation and yet recollection was higher at the fluent as compared to the non-fluent processing.

Thus, although we do not dispute the notion that recollection-based retrieval may benefit from encoding-retrieval compatibility more than familiarity-based retrieval (but see [63], we argue that the extent to which each process would be affected by the manipulation is better determined by the stage in which the manipulation is carried. Our thesis is that the effect on recollection is, for the most part, unrelated to the nature of the manipulation. Rather, it is the stage—encoding versus retrieval—in which the variability of processing the information takes place that primary determines which process would be affected. We propose that given that the fluency manipulation—or for that matter any manipulation at all—was carried out at encoding, it would affect recollection.

## Supporting Information

S1 Table Mean estimates (and SE) of the proportion of Remember and Know responses hit rates, as a function of Fluency (Fluent, Non-fluent) and Memory stage (Encoding, Retrieval). (DOCX)

S2 Table Mean estimates (and SE) of the proportion of Remember and Know responses hit rates, as a function of Attentional: Full, divided at encoding, and divided at retrieval. (DOCX)

S3 Table Mean estimates (and SE) of the proportion of Remember and Know responses hit rates, as a function of Attentional-load (High, Low) and Memory stage (Encoding, Retrieval). (DOCX)
