# Correction: Recollection-Based Retrieval Is Influenced by Contextual Variation at Encoding but Not at Retrieval

**DOI:** 10.1371/journal.pone.0134758

**Published:** 2015-07-31

**Authors:** Eyal Rosenstreich, Yonatan Goshen-Gottstein

The equations in the Experiment 1 section under the subheading “Results and Discussion” are incorrect. The first three paragraphs of that section, containing the corrected equations, are as follows:

We present the data corrected for the estimates of recollection and familiarity: We first calculated, for each participant, the proportion of Remember (R) and Know (K) responses by dividing a given response by the total number of non-guess responses, that is, by the total number of responses excluding guess responses. To illustrate, given that each experimental condition contained 20 items, the R proportion was calculated as: $P\left(R\right)=\frac{R}{20-G}$ where R and G are the raw number of R and G responses. The subtraction of G responses from the total number of trials was performed after post-test debriefing revealed that G responses were mainly due to experimental noise. For example, participants typically responded with G when they pressed the "old" button by mistake, or when they could not decide when an item was old or new. Overall G rates were very small (*M* = .016, *SD* = .035 in the encoding-manipulation block; *M* = .038, *SD* = .071 in the retrieval-manipulation block), and were equally distributed over the experimental conditions (*t*(47) = 0.275, *p* = .785 in the encoding-manipulation block; *F*(3,141) = 0.828, *p* = .480 in the retrieval-manipulation block) (cf., [46]).

Thus, given that each experimental condition contained 20 items, Proportion Rs and Ks were calculated as:

$$P\left(R\right)=\frac{R}{20-G}$$

$$P\left(K\right)=\frac{K}{20-G}$$

where R, K and G are the raw frequencies of R, K and G responses, respectively.

Then, R and K proportions were applied to the correction for the estimates of recollection and familiarity according to Yonelinas and Jacoby's correction formulas [47] [48]. Recollection was estimated by the R proportion: *Recollection* = *P*(*R*) and familiarity was estimated by: $Familiarity=\frac{P\left(K\right)}{1-P\left(R\right)}$

The raw proportions of R and K responses are presented in S1 Table. Effect sizes throughout the article were calculated as Cohen's d.

## References

[1] Rosenstreich E, Goshen-Gottstein Y (2015) Recollection-Based Retrieval Is Influenced by Contextual Variation at Encoding but Not at Retrieval. PLoS ONE 10(7): e0130403 doi:10.1371/journal.pone.0130403 26135583 10.1371/journal.pone.0130403 PMC4489907

