# Supplementary material for: Recollection-Based Retrieval Is Influenced by Contextual Variation at Encoding but Not at Retrieval
Source: PLoS One. 2015 Jul 2;10(7):e0130403. doi: 10.1371/journal.pone.0130403 (PMC4489907; doi:10.1371/journal.pone.0130403)
Supplement: S1 Table — (DOCX) [file pone.0130403.s001.docx]

|  |  | Fluency | | |
| --- | --- | --- | --- | --- |
| Response | Memory stage | Fluent | Non-Fluent | Cohen's *d*^b^ |
| Remember | Encoding | .65 *(.03)* | .58 *(.03)* | 0.34 |
|  | Retrieval | .39 *(.03)* | .42 *(.03)* | -0.14 |
| Know | Encoding | .20 *(.02)* | .25 *(.02)* | -0.36 |
|  | Retrieval | .38 *(.03)* | .30 *(.03)* | 0.38 |

S1 Table

Mean estimates (and SE) of the proportion of Remember and Know responses hit rates^a^, as a function of Fluency (Fluent, Non-fluent) and Memory stage (Encoding, Retrieval).

Note*.* Remember proportions were calculated for each condition as presented in the Result section of Experiment 1. ^a^ False alarms are not presented because they could not be tabulated in all the conditions due to the constraints of the design. ^b^  Cohen's d represents the effect size of the fluency manipulation (for details and interpretation, see [75]).
