# Supplementary material for: Recollection-Based Retrieval Is Influenced by Contextual Variation at Encoding but Not at Retrieval
Source: PLoS One. 2015 Jul 2;10(7):e0130403. doi: 10.1371/journal.pone.0130403 (PMC4489907; doi:10.1371/journal.pone.0130403)
Supplement: S2 Table — (DOCX) [file pone.0130403.s002.docx]

S2 Table

Experiment 2. Mean estimates (and SE) of Remember and Know hit rates, as a function of Attentional: Full, divided at encoding, and divided at retrieval.

|  | Attention manipulation | | | |
| --- | --- | --- | --- | --- |
| Process | Full | Divided at encoding | Divided at retrieval |  |
| Remember | .58 *(.03)* | .22 *(.02)* | .51 *(.03)* |  |
| Know | .29 *(.03)* | .38 *(.02)* | .23 *(.02)* |  |
