# Supplementary material for: Recollection-Based Retrieval Is Influenced by Contextual Variation at Encoding but Not at Retrieval
Source: PLoS One. 2015 Jul 2;10(7):e0130403. doi: 10.1371/journal.pone.0130403 (PMC4489907; doi:10.1371/journal.pone.0130403)
Supplement: S3 Table — (DOCX) [file pone.0130403.s003.docx]

|  |  | Attentional-load | | |
| --- | --- | --- | --- | --- |
| Response | Memory stage | Low | High | Cohen's *d*^b^ |
| Remember | Encoding | .43 *(.05)* | .21 *(.03)* | 1.12 |
|  | Retrieval | .53 *(.05)* | .49 *(.05)* | 0.16 |
| Know | Encoding | .39 *(.04)* | .35 *(.03)* | 0.23 |
|  | Retrieval | .36 *(.05)* | .37 *(.04)* | -0.05 |

S3 Table

Mean estimates (and SE) of the proportion of Remember and Know responses hit rates^a^, as a function of Attentional-load (High, Low) and Memory stage (Encoding, Retrieval).

Note*.* Remember and Know proportions were calculated for each condition as presented in the Result section of Experiment 1. ^a^ False alarms are not presented because they could not be tabulated in all the conditions due to the constraints of the design. ^b^  Cohen's d represents the effect size of the attentional-load manipulation (for details and interpretation, see [75]).
